# Normal Values of Myocardial Deformation Assessed by Cardiovascular Magnetic Resonance Feature Tracking in a Healthy Chinese Population: A Multicenter Study

**DOI:** 10.3389/fphys.2018.01181

**Published:** 2018-09-03

**Authors:** Junping Peng, Xiaodan Zhao, Lei Zhao, Zhanming Fan, Zheng Wang, Hui Chen, Shuang Leng, John Allen, Ru-San Tan, Angela S. Koh, Xiaohai Ma, Mingwu Lou, Liang Zhong

**Affiliations:** ^1^Department of Radiology, The Eighth Affiliated Hospital, Sun Yat-sen University, Shenzhen, China; ^2^Department of Radiology, Beijing Anzhen Hospital, Capital Medical University, Beijing, China; ^3^Post-Doctoral Research Center, Department of Radiology, Longgang Central Hospital, Shenzhen Clinical Medical Institute, Guangzhou University of Chinese Medicine, Shenzhen, China; ^4^National Heart Centre Singapore, Singapore, Singapore; ^5^Duke-NUS Medical School, Singapore, Singapore

**Keywords:** cardiovascular magnetic resonance, feature tracking, strain and deformation, Chinese, multicenter

## Abstract

Reference values on atrial and ventricular strain from cardiovascular magnetic resonance (CMR) are essential in identifying patients with impaired atrial and ventricular function. However, reference values have not been established for Chinese subjects. One hundred and fifty healthy volunteers (75 Males/75 Females; 18–82 years) were recruited. All underwent CMR scans with images acceptable for further strain analysis. Subjects were stratified by age: Group 1, 18–44 years; Group 2, 45–59 years; Group 3, ≥60 years. Feature tracking of CMR cine imaging was used to obtain left atrial global longitudinal (LA *E*_ll_) and circumferential strains (LA *E*_cc_) and respective systolic strain rates, left ventricular longitudinal (LV *E*_ll_), circumferential (LV *E*_cc_) and radial strains (LV *E*_rr_) and their respective strain rates, and right ventricular longitudinal strain (RV *E*_ll_) and strain rate. LA *E*_ll_ and LA *E*_cc_ were 32.8 ± 9.2% and 40.3 ± 13.4%, respectively, and RV *E*_ll_ was −29.3 ± 6.0%. LV *E*_ll_, LV *E*_cc_ and LV *E*_rr_ were −22.4 ± 2.9%, −24.3 ± 3.1%, and 79.0 ± 19.4%, respectively. LV *E*_ll_ and LV *E*_cc_ were higher in females than males (*P* < 0.05). LA *E*_ll_, LA *E*_cc_, and LV *E*_cc_ decreased, while LV *E*_rr_ increased with age (*P* < 0.05). LV *E*_ll_ and RV *E*_ll_ were not shown to be associated with age. Normal ranges for atrial and ventricular strain and strain rates are provided using CMR feature tracking in Chinese subjects.

## Introduction

Assessment of chamber function is an important objective of a cardiac imaging study. In assessing chamber function, myocardial deformation is superior to left ventricular (LV) ejection fraction for prognostication in patients with various myocardial disorders (Stanton et al., 2009; Motoki et al., 2012; Zhong et al., 2013; Kalam et al., 2014). Several advanced techniques based on either echocardiography or cardiovascular magnetic resonance (CMR), such as real-time speckle-tracking echocardiography (Zhang et al., 2016), tissue tagging and feature tracking (Zhong et al., 2012; Venkatesh et al., 2014; Khan et al., 2015), have become available for assessing myocardial deformation. Advantages of these techniques include excellent scan-rescan reproducibility, less dependence on operator technique, and more accurate and reproducible measures of the left and right ventricles. Consequently, CMR has become the standard reference modality for measurement of ventricular volume and function (Bellenger et al., 2000), and arguably the optimal imaging modality for quantification of myocardial displacement (Leng et al., 2015, 2016, 2018), strain and strain rate (Rahman et al., 2017; Scatteia et al., 2017; Koh et al., 2018; Zhao et al., 2018).

Cardiovascular magnetic resonance feature tracking (CMR-FT) is analogous to speckle-tracking echocardiography and allows quantification of global and regional myocardial motion and deformation using standard, balanced steady state free precession (SSFP)/balanced turbo field echo (BTFE) cine CMR images in long and short axis views, which are imperative in routine clinical CMR practice (Augustine et al., 2013). Reference values of cardiac strain for the Western population have been reported in Andre et al. (2015), Kawel-Boehm et al. (2015), Taylor et al. (2015), Mangion et al. (2016). Distribution patterns of LV myocardial strain in healthy Chinese volunteers were provided using deformation registration algorithm (TrufiStrain, Siemens Healthcare), however, strain analysis for left atrium and right ventricle was not performed (Liu et al., 2017).

In this study, we aimed to establish CMR reference values for left atrial (LA), left ventricular (LV), and right ventricular (RV) strains and strain rate in Chinese subjects, and to evaluate the effects of age and gender on strain and strain rate measurements.

## Materials and Methods

### Population

Healthy volunteers who met the following inclusion criteria were prospectively recruited from two centers in China and one in Singapore: (1) no symptoms or prior history of cardiovascular or cerebrovascular disease; (2) no prior diagnosis of hyperlipidaemia, hypertension or diabetes mellitus; (3) normal physical examination and electrocardiogram; (4) no contraindications to CMR. Subjects with wall motion abnormalities or significant valvular diseases detected on CMR were excluded. The study was approved by the local institutional research ethics committee. Written informed consent was obtained from all subjects. The study protocol conforms to the ethical guidelines of the 1975 Declaration of Helsinki as reflected in *a priori* approval by the institution’s human research committee.

### CMR Image Acquisition

Subjects underwent cine CMR on a 1.5T (Verio, Siemens, Germany) or a 3.0T (Prisma, Siemens, Germany) CMR scanners at the China sites and on a 3.0T (3.0T, Ingenia, Philips Healthcare, Netherlands) CMR scanner at the Singapore site, using a standardized imaging protocol (Kramer et al., 2013). Balanced SSFP (China sites) and BTFE (Singapore site) sequences with breath-hold were performed to obtain cine CMR images, comprising a stack of contiguous parallel short-axis slices covering the entire LV and RV from base to apex and three LV long-axis slice (2-, 3-, and 4-chamber views) images. The slice thickness/spacing is 5 mm/1 mm for long axis and 8 mm/2 mm for short axis in China sites; and 8 mm/0 mm for both short and long axis in Singapore site.

### CMR Image Post-processing

Cine CMR image manual segmentation and analyses were conducted by investigators experienced in CMR (JPP, XDZ, and ZW). Minimal and maximal left atrial volumes were calculated at the respective cardiac phases using biplane area-length method (Lang et al., 2005): LA volume (mL) = 0.85^∗^A2C^∗^A4C/L, where A2C and A4C represent planimetered LA area in the 2- and 4-chamber views, respectively; and L, the length of the major LA axis at either the 2- or 4-chamber view, whichever is shorter. LV end-diastolic volume (LVEDV), end-systolic volume (LVESV), stroke volume (LVSV), ejection fraction (LVEF) and LV mass were measured using Qmass (Medis Suite, Netherlands), and applicable values indexed to body surface area were calculated.

### CMR Feature Tracking Analysis

**FIGURE 1:**
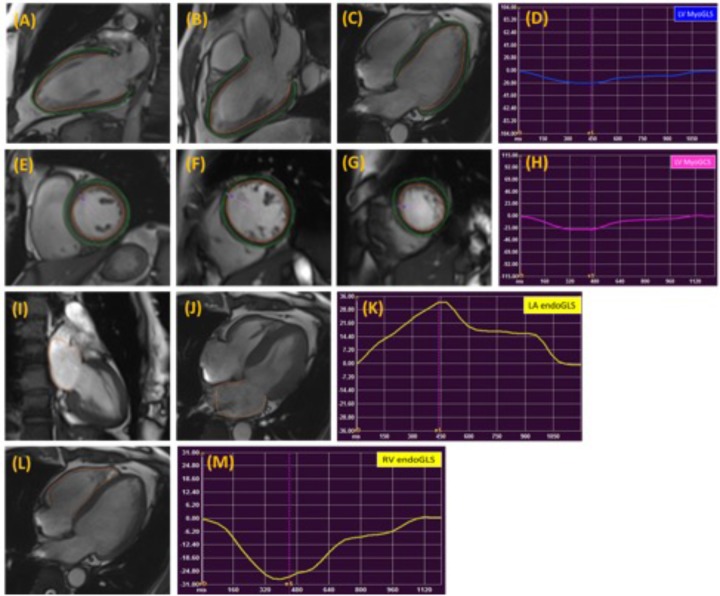
Representative images in the standard long axis **(A–C)** and short axis **(E–G)** orientations and strain curves. Contours are illustrated in left ventricular endocardial and epicardial borders in the 2-, 3-, 4-chamber **(A–C)** and corresponding LV MyoGLS (LV *E*_ll_) **(D)**; endocardial and epicardial borders in base, mid, apical short axis views **(E–G)** and corresponding LV MyoGCS (LV *E*_cc_) **(H)**; left atrial endocardial borders in 2-, and 4-chamber **(I,J)** and corresponding LA endoGLS (LA *E*_ll_) **(K)**; right ventricular endocardial border in 4-chamber **(L)** and corresponding RV endoGLS (RV *E*_ll_) **(M)**.

Global and regional LA, LV, and RV strain and systolic strain rate measurements were analyzed using commercial cardiovascular post-processing software (Medis 3.0, Netherlands). Feature tracking allows quantification of global and regional longitudinal, circumferential and radial strain and strain rates. A short description of the analysis is given here. At end-diastole, endocardial and epicardial borders were manually delineated using a point-and-click approach before the automated tracking algorithm was applied. Papillary muscles included within the endocardial borders (**Figures 1A–C,E–G**). LA endocardial contours were initially traced in the 2- and 4- chamber views at the minimum LA volume after atrial contraction (**Figures 1I,J**). RV endocardial border was traced in 4-chamber view (**Figure 1L**). Guided by signal in homogeneities or anatomical features and using a maximum likelihood method, the software algorithm provides an automatically traced image with frame-to-frame template matching throughout the entire cardiac cycle. Strain values are derived by comparing the movement of the features in relation to each other along the initially drawn borders. CMR feature tracking performance was visually reviewed to ensure accurate tracking. In cases where tracking is determined to be inadequate, the software allows for border editing. Global longitudinal, circumferential and radial strain values (*E*_ll_, *E*_cc_, and *E*_rr_) were automatically extracted from corresponding strain curves (**Figures 1D,H,K,M**).

LA longitudinal strain and strain rate were measured in both 2- and 4-chamber views, excluding the confluence of the pulmonary veins and LA appendage in the border delineation. The LA wall contours were automatically segmented into anterior, inferior, posterior, lateral and atrial septal regions by the software (Kowallick et al., 2014; Zareian et al., 2015).

The standard 17-segment model of the American Heart Association (AHA) (Cerqueira et al., 2002) was applied in the analysis of LV longitudinal strain measured in 2-, 3-, and 4-chamber views. The basal, mid-cavity and apical levels were segmented from the end-diastolic 4-chamber long-axis cine image. For circumferential and radial strain analyses of LV short-axis views, a modified 16-segment model (omitting the apical cap) was applied, using the RV insertion point as the reference point for the junction of LV anterior wall and septum. For short axis strain analysis, base was selected as the slice still showing a complete circumference of myocardium throughout the entire cardiac cycle (without through plane distortion from the LV outflow tract) and the apex was selected as the slice still showing LV cavity at systole (Taylor et al., 2015; Kowallick et al., 2016).

Segmental endocardial and myocardial values of LV were separately measured for longitudinal and circumferential strain and strain rates. To dissect the regional strain distribution, we calculated longitudinal and circumferential strains at basal, middle and apical levels, as well as at the anterior wall, septal, inferior wall and lateral walls of LV by averaging the peak values of the segments corresponding to the relevant territories. For each segment, strain rate was calculated as the derivative of the initial measured strain.

Global LA longitudinal strain (and strain rate) was calculated as the average of strain (and strain rate) from 2-and 4-chamber views, and regional segments were divided as anterior wall, inferior wall, roof, lateral wall and septum wall. The values at roof was taken as the average value from 2- and 4-chamber views. Global and regional RV (free wall and septum) longitudinal strains were measured on the 4-chamber view, and the respective strain rates were calculated (Heermann et al., 2014; Prati et al., 2015).

### Statistical Analysis

All continuous variables were described as mean ± standard deviation (SD). Means of baseline variables among the three age groups were compared using one-way analysis of variance (ANOVA) with *post hoc* pairwise comparisons and the Bonferroni correction for multiple comparisons; comparisons between males and females were performed using a two-sample *t*-test. Correlation was assessed using the Pearson correlation coefficient (*r*). Statistical significance was set at *P* ≤ 0.05. All analyses were performed using SPSS Statistics 22.0. Intra- and inter-observer reproducibility were performed in 20 randomly selected subjects using intra-class coefficient (ICC), Bland–Altman method and the coefficient of variation (CV), which was calculated as the mean of absolute difference between two methods over the mean average.

## Results

### Clinical Characteristics of Study Subjects

One hundred and fifty subjects (75 Males/75 Females, 18–82 years) with cine CMR images acceptable for CMR feature tracking analysis were recruited. A summary of clinical characteristics and global left atrium and left ventricle measurements by age category are presented in **Table 1**. Of the 150 subjects, 84 were from Beijing Anzhen Hospital [40 males, mean (±SD) age 43 ± 12 years], 41 from National Heart Centre Singapore (24 males, mean age 66 ± 8 years), and 25 from Longgang Central Hospital of Shenzhen (12 males; mean age 54 ± 11 years).

**Table 1:** Summary of clinical characteristics and global left atrium and left ventricle measurements by age category.

Variable	All (*n* = 150)	Men				Women			
		Age				Age			
		≤44	45–59	≥ 60	All (*n* = 75)	≤44	45–59	≥60	All (*n* = 75)
Age, years	51 ± 15	33 ± 7	50 ± 4	66 ± 4	50 ± 15	36 ± 5	53 ± 5	68 ± 6	52 ± 14
Weight, kg	65.4 ± 11.2	72.7 ± 10.6	74.7 ± 10.0	64.2 ± 10.7	70.5 ± 11.2	62.9 ± 9.9	61.4 ± 7.3	59.5 ± 6.9	60.3 ± 8.5
Height, cm	165 ± 8	174 ± 5	172 ± 5	165 ± 7	170 ± 7	163 ± 5	159 ± 5	156 ± 5	159 ± 6
Body mass index, kg/m^2^	24.0 ± 2.9	23.9 ± 3.0	25.3 ± 2.8	23.4 ± 3.1	24.2 ± 3	23.6 ± 3.3	24.3 ± 2.4	23.3 ± 2.2	23.7 ± 2.7
Heart rate, bpm	72 ± 14	75 ± 19	70 ± 9	75 ± 16	74 ± 15	72 ± 13	68 ± 9	74 ± 13	71 ± 12
Body surface area, m^2^	1.69 ± 0.18	1.84 ± 0.15	1.85 ± 0.15	1.68 ± 0.16	1.79 ± 0.2	1.64 ± 0.14	1.61 ± 0.11	1.52 ± 0.11	1.59 ± 0.1
LV mass, g	74.6 ± 19.9	86.0 ± 18.6	89.4 ± 16.7	83.8 ± 14.7	86.4 ± 16.7	63.9 ± 18.0	63.0 ± 17.2	61.5 ± 10.2	62.8 ± 15.4
LV EDV, mL	114.2 ± 24.2	131.5 ± 27.0	124.6 ± 19.3	119.6 ± 16.0	125.3 ± 21.6	113.3 ± 24.6	103.5 ± 18.5	92.6 ± 15.8	103.1 ± 21.5
LV ESV, mL	45.8 ± 13.6	54.3 ± 15.1	50.8 ± 10.7	45.5 ± 10.0	50.2 ± 12.5	50.0 ± 14.2	40.7 ± 7.8	33.6 ± 12.0	41.5 ± 13.3
LV SV, mL	68.4 ± 14.8	77.2 ± 15.8	73.9 ± 12.8	74.1 ± 11.7	75 ± 13.5	63.2 ± 13.4	62.8 ± 15.0	59.2 ± 10.6	61.8 ± 13.1
LV EF, %	65 ± 7	65 ± 5	65 ± 7	62 ± 6	65 ± 6	64 ± 5	67 ± 6	65 ± 9	65 ± 7
LV mass index, g/m^2^	44 ± 9	47 ± 9	48 ± 8	50 ± 7	48 ± 8	38 ± 8	39 ± 10.	41 ± 7	40 ± 9
LV EDV index, mL/m^2^	67 ± 11	71 ± 12	67 ± 10	70 ± 9	70 ± 10	69 ± 12	65 ± 10	59 ± 8	64 ± 11
LV ESV index, mL/m^2^	27 ± 7	29 ± 8	27 ± 6	27 ± 6	28 ± 7	30 ± 7	25 ± 4	21 ± 7	26 ± 7
LV SV index, mL/m^2^	40 ± 7	42 ± 7	40 ± 6	43 ± 6	42 ± 7	38 ± 7	39 ± 9	38 ± 6	38 ± 7
Min. LA volume, mL	26.9 ± 9.8	22.1 ± 6.7	29.1 ± 9.1	31.0 ± 9.8	27.4 ± 9.3	22.3 ± 8.5	24.4 ± 7.9	32.4 ± 11.3	26.4 ± 10.2
Max. LA volume, mL	58.6 ± 18.0	52.9 ± 17.1	62.7 ± 17.7	65.2 ± 17.5	60.3 ± 18.0	54.2 ± 17.5	52.4 ± 16.5	64.4 ± 18.5	57.0 ± 18.1
LAEF, %	58 ± 9	61 ± 7	58 ± 7	54 ± 7	58 ± 8	63 ± 7	62 ± 8	52 ± 10	59 ± 10
Min. LA volume index, mL/m^2^	16.0 ± 6.0	11.9 ± 3.1	15.8 ± 4.9	18.4 ± 5.3	15.4 ± 5.2	13.5 ± 5.0	15.2 ± 4.8	21.4 ± 7.3	16.7 ± 6.6
Max. LA volume index, mL/m^2^	34.8 ± 10.6	28.7 ± 9.0	34.0 ± 9.6	38.8 ± 9.4	33.9 ± 10.1	32.8 ± 10.2	32.6 ± 9.9	42.2 ± 11.0	35.9 ± 11.2

*Data are expressed as mean ± SD or as number (percentage). LV, left ventricular; EDV, end-diastolic volume; ESV, end-systolic volume; SV, stroke volume; EF, ejection fraction; LA, left atrial; Min. LA volume, minimal left atrial volume; Max. LA volume, maximal left atrial volume. The LA EDV, LA ESV and LA EF were from Medis software. Min. LA volume and Max. LA volume were volumes using biplane area-length method.*

### Global and Regional Strain Values

Normal values for global LA endocardial, LV myocardial and RV endocardial CMR feature-tracking deformation measured parameters are shown in **Table 2A**. Mean endocardial LA *E*_ll_ and LA *E*_cc_ were 32.8 ± 9.2 and 40.3 ± 13.4%, respectively. For LA *E*_ll_ at the segmental level, the LA roof exhibited the lowest strain (30.7 ± 13.9%), and differences between the LA roof and LA *E*_ll_ at the anterior (38.7 ± 18.5%) and inferior (41.3 ± 20.4%) walls were both significant (*P* ≤ 0.003). LA strain rate at the anterior wall (1.66 ± 0.89 s^−1^) was significantly higher than at the LA roof (1.35 ± 0.52 s^−1^), lateral wall (1.30 ± 0.63 s^−1^) and septal wall (1.40 ± 0.61 s^−1^) (all *P* ≤ 0.01). Age and gender specific global LA endocardial, LV myocardial and RV endocardial strain values are given in **Table 2B**.

**Table 2A:** Age and gender global strains of LA endocardium, LV myocardium and RV endocardium.

		All (*n* = 150)	Age				Gender		
			≤44 (*n* = 50)	45–59 (*n* = 50)	≥60 (*n* = 50)	*P*	Male (*n* = 75)	Female (*n* = 75)	*P*
LA	*E*_ll_, %	32.8 ± 9.2	36.0 ± 8.7	33.9 ± 8.7	28.7 ± 8.9^∗#^	<0.001	32.3 ± 9.5	33.3 ± 9.0	0.505
	*E*_cc_, %	40.3 ± 13.4	45.0 ± 14.7	42.8 ± 12.2	33.1 ± 10.1^∗#^	<0.001	39.5 ± 12.1	41.1 ± 14.7	0.473
LV	*E*_ll_, %	−22.4 ± 2.9	−23.0 ± 2.7	−22.4 ± 3.1	−20.9 ± 2.7	0.155	−21.6 ± 2.5	−23.3 ± 2.9	<0.001
	*E*_cc_, %	−24.3 ± 3.1	−25.0 ± 2.8	−24.2 ± 3.2	−23.6 ± 3.2	0.060	−23.7 ± 3.1	−24.9 ± 3.1	0.016
	*E*_rr_, %	79.0 ± 19.4	72.9 ± 15.9	78.1 ± 20.8^∗^	86.3 ± 19.2^∗#^	0.002	76.2 ± 17.9	81.9 ± 20.6	0.075
RV	*E*_ll_, %	−29.3 ± 6.0	−30.0 ± 6.1	−29.1 ± 6.7	−28.8 ± 5.0	0.559	−28.5 ± 6.4	−30.1 ± 5.5	0.104

*Data are expressed as mean ± SD. LA, left atrial; LV, left ventricle; RV, right ventricle; E_ll_, global longitudinal strain; E_cc_, global circumferential strain; E_rr_, global radial strain. ^∗^Difference between the young and the old was significant (P < 0.05). ^#^Difference between the middle aged and the old was significant (P < 0.05).*

**Table 2B:** Age and gender global strains of LA endocardium, LV myocardium and RV endocardium.

		≤44			45–59			≥60		
		Male (*n* = 25)	Female (*n* = 25)	*P*	Male (*n* = 25)	Female (*n* = 25)	*P*	Male (*n* = 25)	Female (*n* = 25)	*P*
LA	*E*_ll_, %	35.5 ± 9.6	36.4 ± 7.8	0.738	31.8 ± 8.5	35.9 ± 8.5	0.096	29.6 ± 9.7	27.7 ± 8.0	0.454
	*E*_cc_, %	44.0 ± 15.3	45.9 ± 14.4	0.651	40.7 ± 9.8	44.8 ± 14.1	0.235	33.7 ± 7.9	32.5 ± 12.0	0.647
LV	*E*_ll_, %	−22.4 ± 2.0	−23.6 ± 3.1	0.119	−21.4 ± 3	−23.3 ± 2.8	0.021^∗^	−20.9 ± 2.2	−22.9 ± 2.9	0.009*^#^*
	*E*_cc_, %	−25.4 ± 2.9	−24.7 ± 2.8	0.415	−23.3 ± 3.1	−25.1 ± 3	0.040^∗^	−22.3 ± 2.4	−24.8 ± 3.5	0.005*^#^*
	*E*_rr_, %	73.7 ± 13.9	71.9 ± 18.0	0.687	72.5 ± 19.5	83.6 ± 20.8	0.059	82.4 ± 18.6	90.2 ± 19.4	0.156
RV	*E*_ll_, %	−30.6 ± 6.1	−29.4 ± 6.2	0.482	−24.5 ± 14.2	−28.3 ± 13.7	0.334	−27.8 ± 4.0	−29.8 ± 5.8	0.164

*^∗^Difference between the middle-aged men and women was significant (P < 0.05). ^#^Difference between the old men and women was significant (P < 0.05).*

**FIGURE 2:**
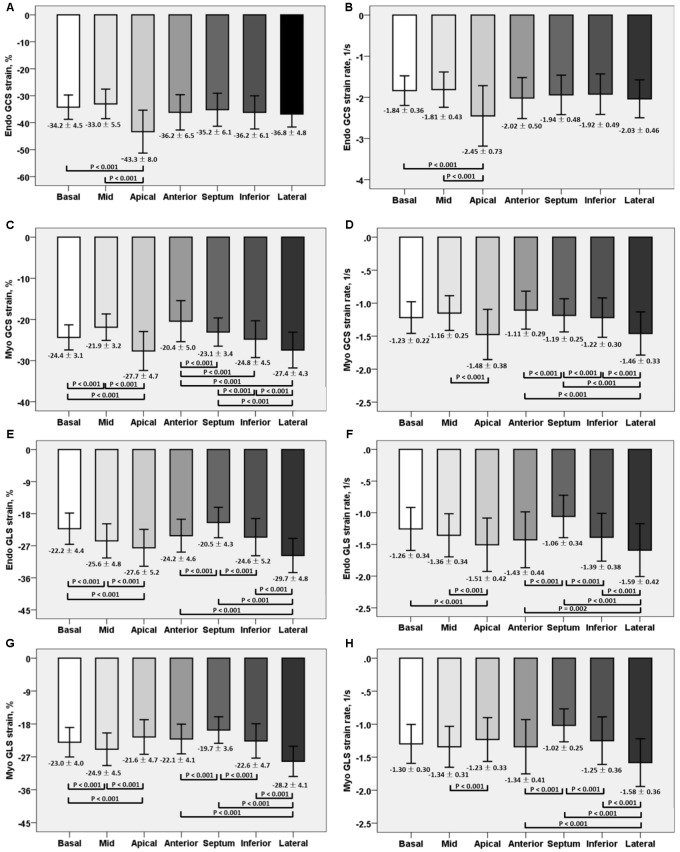
Comparison of endocardial circumferential strain and strain rate **(A)** and **(B)**; myocardial circumferential strain and strain rate **(C)** and **(D)**; endocardial longitudinal strain and strain rate **(E)** and **(F)**; and myocardial longitudinal strain and strain rate **(G)** and **(H)** at basal, mid and apical levels; anterior, septum, inferior and lateral walls.

For LV myocardium strains, mean LV *E*_ll_, LV *E*_cc_ and LV *E*_rr_ was −22.4 ± 2.9, −24.3 ± 3.1, and 79.0 ± 19.4%, respectively. The endocardial and myocardial circumferential strain and strain rates for regional 16 segments are provided in **Table 3**. Barchart plots (with one standard deviation) for segmental LV endocardial and myocardial circumferential strains and strain rates at basal, mid-cavity and apical levels, and at the anterior, septum, inferior and lateral walls are given in **Figures 2A–D**. Means for both endocardial and myocardial LV *E*_cc_ were significantly higher at the apical level than at the base and mid-cavity levels (all *P* < 0.001), and the mid-level exhibited the lowest strain among the three levels (*P* < 0.001). For both endocardial and myocardial circumferential strain rates, significantly larger values were observed at the apical level compared to mid-cavity (*P* < 0.001). Furthermore, myocardial *E*_cc_ and strain rate increased significantly from anterior → septum → inferior → lateral walls (all *P* < 0.001), while no differences were found for endocardial *E*_cc_ and strain rate among these four regional walls. In contrast, endocardial LV *E*_ll_ increased significantly from basal to mid to apex (**Figure 2E**), and mean myocardial LV *E*_ll_ was higher at the middle level rather than at the basal and apical levels (**Figure 2G**). Lateral walls had significantly higher endocardial and myocardial *E*_ll_ and strain rates than anterior walls, and inferior and septum walls, with septum walls having the lowest strain and strain rates (**Figures 2F,H**). Endocardial and myocardial longitudinal strain and strain rates for the regional 17 segments are provided in **Table 4**.

**Table 3:** Left ventricular segmental endocardial and myocardial circumferential strain and strain rates from short axis view.

Segment	Circumferential endocardial strain, %	Circumferential endocardial strain rate, s^−1^	Circumferential myocardial strain, %	Circumferential myocardial strain rate, s^−1^
1	−33.2 ± 8.9	−1.80 ± 0.55	−21.1 ± 6.8	−1.12 ± 0.37
2	−33.9 ± 8.5	−1.87 ± 0.61	−20.7 ± 6.4	−1.07 ± 0.37
3	−31.1 ± 8.4	−1.63 ± 0.55	−23.6 ± 6.1	−1.16 ± 0.38
4	−31.4 ± 7.6	−1.58 ± 0.49	−20.8 ± 6.5	−0.94 ± 0.33
5	−37.3 ± 6.3	−2.07 ± 0.55	−28.8 ± 6.5	−1.46 ± 0.41
6	−38.6 ± 7.2	−2.08 ± 0.58	−31.3 ± 7.3	−1.61 ± 0.50
7	−34.0 ± 8.6	−1.92 ± 0.63	−18.4 ± 6.6	−1.01 ± 0.35
8	−34.6 ± 9.5	−1.96 ± 0.71	−19.7 ± 6.5	−1.07 ± 0.43
9	−32.3 ± 8.6	−1.75 ± 0.58	−23.7 ± 5.4	−1.18 ± 0.33
10	−31.8 ± 7.2	−1.66 ± 0.46	−21.5 ± 5.8	−1.07 ± 0.32
11	−36.0 ± 6.4	−1.98 ± 0.57	−25.5 ± 6.2	−1.36 ± 0.46
12	−29.6 ± 9.0	−1.60 ± 0.58	−22.6 ± 9.1	−1.25 ± 0.43
13	−41.3 ± 9.2	−2.34 ± 0.79	−21.8 ± 8.0	−1.19 ± 0.48
14	−44.1 ± 9.6	−2.48 ± 0.84	−27.6 ± 7.1	−1.44 ± 0.44
15	−45.3 ± 9.9	−2.53 ± 0.86	−32.1 ± 7.2	−1.65 ± 0.53
16	−42.5 ± 9.6	−2.45 ± 0.85	−29.1 ± 7.6	−1.63 ± 0.52

*Data are expressed as mean ± SD. 1, basal anterior; 2, basal anterolateral; 3, basal inferoseptal; 4, basal inferior; 5, basal inferolateral; 6, basal anterolateral; 7, mid anterior; 8, mid anteroseptal; 9, mid inferoseptal; 10, mid inferior; 11, mid inferolateral; 12, mid anterolateral; 13, apical anterior; 14, apical septal; 15, apical inferior; 16, apical lateral.*

**Table 4:** Left ventricular segmental endocardial and myocardial longitudinal strain and strain rates from long axis view.

Segment	Longitudinal endocardial strain, %	Longitudinal endocardial strain rate, s^−1^	Longitudinal myocardial strain, %	Longitudinal myocardial strain rate, s^−1^
1	−14.8 ± 7.8	−1.01 ± 0.58	−12.6 ± 7.5	−0.98 ± 0.59
2	−15.3 ± 9.1	−0.93 ± 0.46	−19.0 ± 8.1	−1.03 ± 0.44
3	−18.8 ± 6.9	−0.88 ± 0.55	−18.1 ± 6.3	−0.88 ± 0.32
4	−20.1 ± 8.8	−1.29 ± 0.60	−22.1 ± 8.8	−1.30 ± 0.60
5	−32.5 ± 8.0	−1.79 ± 0.53	−33.4 ± 8.8	−1.84 ± 0.58
6	−31.7 ± 8.6	−1.64 ± 0.68	−32.9 ± 8.8	−1.77 ± 0.59
7	−23.9 ± 10.8	−1.41 ± 0.63	−23.1 ± 10.8	−1.35 ± 0.61
8	−22.9 ± 7.9	−1.22 ± 0.47	−20.7 ± 7.8	−1.10 ± 0.44
9	−23.7 ± 8.0	−1.11 ± 0.59	−24.0 ± 7.4	−1.17 ± 0.44
10	−22.5 ± 9.8	−1.20 ± 0.49	−23.5 ± 9.4	−1.23 ± 0.51
11	−31.0 ± 8.8	−1.62 ± 0.60	−29.7 ± 10.4	−1.62 ± 0.65
12	−29.8 ± 9.8	−1.57 ± 0.73	−28.5 ± 10.5	−1.59 ± 0.67
13	−33.9 ± 8.9	−1.86 ± 0.69	−30.7 ± 9.9	−1.70 ± 0.66
14	−21.7 ± 6.7	−1.16 ± 0.42	−16.6 ± 5.7	−0.91 ± 0.32
15	−31.1 ± 9.6	−1.67 ± 0.64	−22.3 ± 10.7	−1.22 ± 0.53
16	−23.6 ± 8.5	−1.33 ± 0.52	−16.6 ± 7.9	−1.10 ± 0.43
17	−31.6 ± 5.9	−1.69 ± 0.50	−23.9 ± 5.6	−1.26 ± 0.40

*Data are expressed as mean ± SD. 1, basal anterior; 2, basal anterolateral; 3, basal inferoseptal; 4, basal inferior; 5, basal inferolateral; 6, basal anterolateral; 7, mid anterior; 8, mid anteroseptal; 9, mid inferoseptal; 10, mid inferior; 11, mid inferolateral; 12, mid anterolateral; 13, apical anterior; 14, apical septal; 15, apical inferior; 16, apical lateral; 17, apex.*

Mean RV *E*_ll_ was −29.3 ± 6.0%, with RV free wall exhibiting greater longitudinal strain (-35.3 ± 7.4% vs. −23.9 ± 6.9%) and strain rate (-1.83 ± 0.46 s^−1^ vs. −1.12 ± 0.39 s^−1^) than the septum (both *P* < 0.001).

### Age and Gender Difference in Global and Regional Strain and Strain Rate

Global strain values for LA, LV, and RV among age groups is given in **Table 2**. For age groups 1, 2, and 3, respectively, global strain values were 36.0 ± 8.7, 33.9 ± 8.7, and 28.7 ± 8.9% for LA *E*_ll_, and 45.0 ± 14.7, 42.8 ± 12.2, and 33.1 ± 10.1% for LA *E*_cc_. LA *E*_ll_ and *E*_cc_ decreased in magnitude with increasing age; correlations were statistically significant but weak (*r* = −0.330 for LA *E*_ll_ and *r* = −0.364 for LA *E*_cc_). LA endocardial strain differences between male and female were non-significant, with values of 32.3 ± 9.5% vs. 33.3 ± 8.9% for *E*_ll_ (*P* = 0.505) and 39.5 ± 12.1% vs. 41.1 ± 14.7% for *E*_cc_ (*P* = 0.473).

In age groups 1, 2, and 3, mean values for LV myocardial *E*_ll_ were −23.0 ± 2.7, −22.4 ± 3.1, and −20.9 ± 2.7; for LV *E*_cc_ −25.0 ± 2.8, −24.2 ± 3.2, and −23.6 ± 3.2%; and for LV *E*_rr_ 72.8 ± 15.9, 78.1 ± 20.8, and 86.3 ± 19.2%. Linear regression showed weak but statistically significant associations, respectively, of *E*_cc_ and *E*_rr_ with age (*r* = 0.208 and *r* = 0.258, both *P* < 0.05). Differences between males and females were significant for LV *E*_ll_ (-21.6 ± 2.5% vs. −23.3 ± 2.9%) and LV *E*_cc_ (-23.7 ± 3.1% vs. −24.9 ± 3.1%) (both *P* < 0.05). Difference in LV *E*_rr_ between genders (76.2 ± 17.9% vs. 81.9 ± 20.6%) was non-significant (*P* = 0.075). Comparison of male and female for three age groups with *P*-value was tabulated in **Table 2B**. Age and gender specific endocardial and myocardial *E*_cc_ values for 16 segments are given in **Tables 5**, **6**, and endocardial and myocardial *E*_ll_ for 17 segments are tabulated in **Tables 7**, **8**.

**Table 5:** Left ventricular circumferential endocardial peak systolic strain values from short axis view.

Segment	Men				Women			
	Age				Age			
	≤44	45–59	≥60	All	≤44	45–59	≥60	All
1	−31.1 ± 6.9	−30.9 ± 6.8	−34.5 ± 6.0	−32.2 ± 6.7	−30.5 ± 8.6	−35.8 ± 11.1	−36.3 ± 11.2	−34.2 ± 10.6
2	−34.7 ± 9.3	−31.7 ± 7.5	−32.2 ± 9.5	−32.9 ± 8.8	−34.2 ± 6.9	−35.4 ± 6.9	−35.2 ± 10.4	−34.9 ± 8.1
3	−26.6 ± 8.4	−31.1 ± 6.2	−32.5 ± 7.1	−30.1 ± 7.6	−28.5 ± 8.2	−29.6 ± 7.0	−38.2 ± 8.9	−32.1 ± 9.1
4	−30.1 ± 5.6	−32.6 ± 6.5	−27.7 ± 8.6	−30.2 ± 7.2	−31.3 ± 7.4	−33.1 ± 8.3	−33.3 ± 8.3	−32.6 ± 7.9
5	−36.9 ± 5.2	−36.0 ± 5.6	−34.5 ± 5.5	−35.8 ± 5.4	−38.2 ± 6.0	−40.0 ± 5.9	−38.7 ± 8.4	−38.8 ± 6.8
6	−38.1 ± 6.2	−34.9 ± 7.8	−36.3 ± 5.7	−36.4 ± 6.7	−41.3 ± 6.5	−40.8 ± 5.2	−40.5 ± 8.9	−40.9 ± 7.0
7	−36.9 ± 5.9	−33.3 ± 8.9	−34.5 ± 6.0	−34.2 ± 7.5	−32.3 ± 9.6	−31.7 ± 8.3	−37.4 ± 10.3	−33.8 ± 9.7
8	−33.2 ± 7.6	−34.8 ± 9.2	−33.8 ± 9.4	−33.9 ± 8.7	−30.2 ± 9.8	−36.2 ± 10.8	−39.4 ± 8.0	−35.2 ± 10.2
9	−29.7 ± 7.9	−33.1 ± 8.1	−34.6 ± 8.6	−32.5 ± 8.4	−27.5 ± 8.8	−32.8 ± 7.0	−35.9 ± 8.9	−32.1 ± 8.9
10	−29.3 ± 6.5	−32.4 ± 7.9	−30.1 ± 7.6	−30.6 ± 7.4	−31.9 ± 7.6	−32.1 ± 6.9	−35.0 ± 6.2	−33.0 ± 7.0
11	−37.6 ± 4.9	−35.6 ± 6.5	−33.0 ± 6.5	−35.4 ± 6.3	−35.2 ± 6.0	−37.8 ± 5.8	−36.5 ± 7.7	−36.5 ± 6.5
12	−30.9 ± 6.4	−30.7 ± 10.3	−27.1 ± 8.8	−29.6 ± 8.7	−28.8 ± 8.4	−29.8 ± 9.8	−30.8 ± 10.1	−29.7 ± 9.4
13	−44.5 ± 10.8	−41.3 ± 10.3	−38.5 ± 7.1	−38.5 ± 7.1	−40.7 ± 9.6	−39.3 ± 8.0	−43.5 ± 8.1	−41.1 ± 8.7
14	−43.8 ± 10.1	−45.0 ± 8.9	−43.5 ± 6.9	−44.1 ± 8.7	−40.3 ± 12.3	−45.6 ± 10.1	−46.6 ± 7.7	−44.2 ± 10.4
15	−49.5 ± 10.8	−46.1 ± 9.0	−40.6 ± 9.8	−45.4 ± 10.5	−43.7 ± 11.2	−46.0 ± 9.0	−46.2 ± 8.2	−45.3 ± 9.5
16	−49.2 ± 9.6	−42.7 ± 9.6	−38.3 ± 8.6	−43.4 ± 10.2	−42.1 ± 9.4	−42.0 ± 7.5	−40.8 ± 10.1	−41.6 ± 9.0
Basal	−32.9 ± 3.6	−32.9 ± 3.4	−33.0 ± 3.9	−32.9 ± 3.6	−34.0 ± 4.6	−35.7 ± 4.1	−37.0 ± 5.7	−35.6 ± 5.0
Mid	−32.9 ± 4.0	−33.3 ± 6.4	−31.8 ± 4.8	−32.7 ± 5.1	−31.0 ± 5.4	−33.4 ± 5.6	−35.8 ± 5.7	−33.4 ± 5.8
Apical	−46.7 ± 9.0	−43.8 ± 8.5	−40.2 ± 8.6	−43.6 ± 8.4	−41.7 ± 8.7	−43.2 ± 7.0	−44.2 ± 6.7	−43.0 ± 7.5

*Data are expressed as mean ± SD.*

**Table 6:** Left ventricular circumferential myocardial peak systolic strain values from short axis view.

Segment	Men				Women			
	Age				Age			
	≤44	45–59	≥60	All	≤44	45–59	≥60	All
1	−20.3 ± 6.4	−19.0 ± 5.6	−22.0 ± 6.1	−20.5 ± 6.1	−20.7 ± 6.0	−21.3 ± 8.9	−22.9 ± 7.0	−21.6 ± 7.4
2	−21.7 ± 6.0	−17.6 ± 6.1	−20.2 ± 7.2	−19.8 ± 6.6	−21.3 ± 5.1	−20.9 ± 6.2	−22.6 ± 6.7	−21.6 ± 6.0
3	−20.7 ± 6.3	−22.6 ± 5.2	−24.0 ± 6.1	−22.4 ± 6.0	−23.8 ± 7.0	−23.8 ± 5.2	−27.0 ± 5.3	−24.9 ± 6.0
4	−19.5 ± 5.9	−21.6 ± 7.0	−19.5 ± 5.0	−20.2 ± 6.0	−21.9 ± 6.6	−22.3 ± 5.8	−19.8 ± 8.3	−21.3 ± 7.0
5	−28.2 ± 4.7	−26.6 ± 5.9	−27.3 ± 6.1	−27.4 ± 5.6	−30.7 ± 5.1	−30.4 ± 6.0	−29.5 ± 9.3	−30.2 ± 7.0
6	−31.9 ± 6.4	−27.4 ± 8.2	−28.7 ± 4.7	−29.3 ± 6.8	−35.0 ± 7.5	−32.7 ± 7.1	−31.8 ± 7.3	−33.2 ± 7.3
7	−19.4 ± 6.8	−17.3 ± 5.5	−19.7 ± 5.9	−18.8 ± 6.1	−18.1 ± 7.0	−15.0 ± 6.1	−20.9 ± 7.1	−18.0 ± 7.1
8	−19.2 ± 5.7	−19.1 ± 7.2	−18.2 ± 5.3	−18.9 ± 6.0	−18.8 ± 6.4	−19.9 ± 7.5	−22.7 ± 6.2	−20.5 ± 6.9
9	−23.8 ± 3.5	−23.8 ± 5.1	−23.2 ± 5.4	−23.6 ± 4.7	−22.3 ± 5.6	−25.0 ± 5.8	−24.0 ± 6.5	−23.8 ± 6.0
10	−18.9 ± 5.8	−21.8 ± 5.0	−22.3 ± 6.3	−21.0 ± 5.8	−21.3 ± 5.4	−21.2 ± 5.6	−23.6 ± 6.3	−22.0 ± 5.8
11	−27.7 ± 4.9	−24.0 ± 6.6	−22.1 ± 6.3	−24.6 ± 6.3	−27.0 ± 5.6	−27.5 ± 4.6	−24.5 ± 7.0	−26.3 ± 5.9
12	−23.4 ± 7.0	−21.9 ± 9.6	−19.4 ± 9.1	−21.6 ± 8.7	−21.7 ± 8.9	−24.5 ± 11.4	−24.7 ± 7.9	−23.6 ± 9.5
13	−25.2 ± 10.2	−21.3 ± 7.4	−20.6 ± 5.9	−22.3 ± 8.2	−22.1 ± 8.4	−18.6 ± 8.2	−23.3 ± 5.9	−21.3 ± 7.8
14	−27.5 ± 6.0	−26.0 ± 6.3	−27.3 ± 4.5	−26.9 ± 5.6	−26.4 ± 8.0	−30.3 ± 7.4	−28.2 ± 9.5	−28.3 ± 8.4
15	−34.9 ± 7.2	−32.5 ± 5.5	−29.1 ± 7.4	−32.1 ± 7.1	−32.0 ± 8.5	−32.4 ± 7.4	−31.8 ± 6.5	−32.1 ± 7.4
16	−34.7 ± 5.8	−28.8 ± 7.1	−23.2 ± 5.7	−28.9 ± 7.8	−30.8 ± 6.7	−31.1 ± 7.4	−26.2 ± 7.8	−29.4 ± 7.6
Basal	−23.7 ± 2.7	−22.5 ± 2.3	−23.6 ± 2.5	−23.3 ± 2.5	−25.5 ± 3.0	−25.0 ± 2.8	−25.6 ± 3.7	−25.4 ± 3.2
Mid	−22.1 ± 2.4	−21.3 ± 3.2	−20.8 ± 3.3	−21.4 ± 3.0	−21.6 ± 2.9	−22.2 ± 3.6	−23.4 ± 3.6	−22.4 ± 3.4
Apical	−30.6 ± 5.2	−27.1 ± 4.8	−25.0 ± 3.4	−27.6 ± 5.0	−27.8 ± 4.6	−28.1 ± 4.7	−27.4 ± 4.2	−27.8 ± 4.5

*Data are expressed as mean ± SD.*

**Table 7:** Left ventricular longitudinal endocardial peak systolic strain values from long axis view.

Segment	Men				Women			
	Age				Age			
	≤44	45–59	≥60	All	≤44	45–59	≥60	All
1	−17.4 ± 7.7	−12.9 ± 7.0	−15.6 ± 7.9	−15.3 ± 7.7	−15.6 ± 8.4	−14.6 ± 8.0	−12.6 ± 7.5	−14.2 ± 8.0
2	−18.5 ± 7.6	−13.8 ± 5.6	−9.4 ± 8.2	−13.9 ± 8.0	−20.7 ± 8.9	−18.4 ± 7.2	−11.1 ± 10.8	−16.7 ± 9.9
3	−20.9 ± 5.5	−17.5 ± 5.4	−20.7 ± 6.0	−19.7 ± 5.8	−18.5 ± 4.6	−20.3 ± 8.6	−14.8 ± 8.6	−17.9 ± 7.8
4	−17.2 ± 7.6	−18.9 ± 8.6	−20.8 ± 7.4	−19.0 ± 8.0	−22.3 ± 8.5	−24.8 ± 8.3	−16.6 ± 10.0	−21.3 ± 9.5
5	−33.3 ± 7.6	−30.1 ± 8.6	−30.9 ± 6.7	−31.4 ± 7.7	−32.8 ± 6.5	−35.6 ± 8.7	−32.6 ± 9.3	−33.7 ± 8.3
6	−28.9 ± 7.7	−31.3 ± 7.5	−28.7 ± 8.6	−29.6 ± 7.9	−33.1 ± 7.7	−32.7 ± 10.2	−35.9 ± 8.3	−33.9 ± 8.8
7	−17.7 ± 7.2	−22.1 ± 9.9	−23.8 ± 11.9	−21.2 ± 10.1	−27.2 ± 10.0	−26.2 ± 10.7	−26.2 ± 12.2	−26.6 ± 10.9
8	−22.6 ± 8.8	−21.8 ± 8.3	−21.1 ± 8.1	−21.9 ± 8.3	−25.0 ± 6.9	−25.0 ± 5.0	−22.0 ± 9.7	−24.0 ± 7.5
9	−23.3 ± 7.2	−23.0 ± 9.2	−23.2 ± 6.7	−23.1 ± 7.6	−25.0 ± 6.8	−25.5 ± 8.3	−22.4 ± 9.6	−24.3 ± 8.3
10	−23.6 ± 9.8	−20.5 ± 6.9	−18.8 ± 11.0	−20.9 ± 9.5	−25.9 ± 10.0	−27.8 ± 9.0	−18.5 ± 8.6	−24.1 ± 10.0
11	−28.4 ± 8.6	−31.5 ± 6.7	−26.1 ± 7.8	−28.7 ± 7.9	−33.7 ± 8.3	−34.5 ± 8.4	−31.7 ± 10.2	−33.3 ± 9.0
12	−25.5 ± 11.6	−28.2 ± 9.5	−27.9 ± 8.0	−27.2 ± 9.8	−31.3 ± 7.9	−31.5 ± 11.1	−34.1 ± 8.5	−32.3 ± 9.2
13	−32.9 ± 7.4	−32.0 ± 7.3	−30.2 ± 9.5	−31.7 ± 8.1	−34.5 ± 8.9	−35.9 ± 9.5	−37.6 ± 9.0	−36.0 ± 9.1
14	−23.6 ± 5.5	−21.2 ± 7.9	−20.1 ± 5.0	−21.6 ± 6.4	−21.9 ± 8.2	−19.3 ± 6.7	−24.0 ± 5.6	−21.7 ± 7.1
15	−34.3 ± 8.1	−30.2 ± 10.7	−28.7 ± 9.5	−31.1 ± 9.7	−32.6 ± 9.4	−27.6 ± 10.1	−33.4 ± 8.6	−31.2 ± 9.6
16	−26.2 ± 7.3	−25.8 ± 9.1	−22.4 ± 4.7	−24.8 ± 7.3	−23.7 ± 9.9	−20.7 ± 10.5	−23.0 ± 7.7	−22.5 ± 9.4
17	−35.3 ± 5.3	−31.7 ± 5.4	−29.7 ± 5.6	−32.2 ± 5.9	−30.9 ± 6.6	−29.2 ± 5.8	−32.8 ± 5.2	−31.0 ± 6.0
Basal	−22.7 ± 2.7	−20.7 ± 3.3	−21.0 ± 4.3	−21.5 ± 3.5	−23.9 ± 3.7	−24.4 ± 5.2	−20.6 ± 5.3	−22.9 ± 5.0
Mid	−23.5 ± 3.9	−24.5 ± 4.3	−23.5 ± 4.7	−23.8 ± 4.3	−28.0 ± 3.0	−28.4 ± 4.4	−25.8 ± 5.8	−27.4 ± 4.6
Apical	−29.2 ± 4.2	−27.3 ± 5.5	−25.3 ± 4.5	−27.3 ± 5.0	−28.2 ± 5.9	−25.9 ± 5.7	−29.5 ± 4.1	−27.8 ± 5.4
Anterior	−22.7 ± 3.9	−22.4 ± 4.2	−23.2 ± 4.3	−22.7 ± 4.1	−25.8 ± 4.7	−25.6 ± 5.0	−25.5 ± 4.5	−25.6 ± 4.7
Septum	−21.8 ± 3.2	−19.5 ± 3.6	−18.9 ± 4.0	−20.0 ± 3.8	−22.2 ± 2.7	−21.7 ± 4.3	−18.9 ± 6.0	−20.9 ± 4.7
Inferior	−25.0 ± 5.0	−23.0 ± 4.4	−22.8 ± 5.1	−23.7 ± 4.9	−26.9 ± 6.5	−26.7 ± 3.9	−22.8 ± 4.7	−25.5 ± 5.4
Lateral	−28.5 ± 4.0	−29.4 ± 5.2	−27.2 ± 3.7	−28.3 ± 4.4	−30.9 ± 3.0	−31.0 ± 5.6	−31.4 ± 5.6	−31.1 ± 4.8

*Data are expressed as mean ± SD. 1, basal anterior; 2, basal anterolateral; 3, basal inferoseptal; 4, basal inferior; 5, basal inferolateral; 6, basal anterolateral; 7, mid anterior; 8, mid anteroseptal; 9, mid inferoseptal; 10, mid inferior; 11, mid inferolateral; 12, mid anterolateral; 13, apical anterior; 14, apical septal; 15, apical inferior; 16, apical lateral; 17, apex. Basal, segments 1–6; Mid, segments 7–12; Apical, segments 13–16; Anterior, segments 1, 7, 13; Septum, segments 2, 3, 8, 9, 14; Inferior, segments 4, 10, 15; Lateral, segments 5, 6, 11, 12, 16.*

**Table 8:** Left ventricular longitudinal myocardial peak systolic strain values from long axis view.

Segment	Men				Women			
	Age				Age			
	≤44	45–59	≥60	All	≤44	45–59	≥60	All
1	−14.7 ± 7.2	−9.9 ± 5.5	−13.3 ± 8.5	−12.7 ± 7.4	−14.7 ± 8.9	−12.4 ± 7.4	−10.3 ± 6.4	−12.5 ± 7.8
2	−20.5 ± 7.4	−17.3 ± 6.7	−14.6 ± 6.9	−17.5 ± 7.3	−23.3 ± 7.8	−22.2 ± 5.7	−15.8 ± 10.1	−20.4 ± 8.6
3	−19.6 ± 4.9	−16.5 ± 6.4	−18.8 ± 6.2	−18.3 ± 6.0	−17.1 ± 4.7	−20.7 ± 6.8	−15.7 ± 7.3	−17.8 ± 6.6
4	−17.8 ± 6.7	−18.3 ± 8.8	−25.1 ± 7.2	−20.4 ± 8.2	−23.1 ± 9.0	−25.7 ± 8.4	−22.7 ± 9.7	−23.8 ± 9.0
5	−32.0 ± 8.2	−30.0 ± 10.2	−33.9 ± 6.6	−31.9 ± 8.5	−33.2 ± 8.1	−36.1 ± 9.4	−35.2 ± 9.2	−34.9 ± 8.9
6	−28.3 ± 7.9	−34.0 ± 8.7	−30.7 ± 8.5	−30.9 ± 8.6	−32.5 ± 8.0	−36.0 ± 7.7	−36.2 ± 9.6	−34.9 ± 8.5
7	−17.9 ± 8.3	−21.4 ± 10.5	−23.7 ± 11.4	−21.0 ± 10.6	−28.3 ± 9.3	−23.8 ± 11.4	−23.7 ± 11.8	−25.3 ± 11.0
8	−19.6 ± 9.2	−19.3 ± 7.8	−21.6 ± 7.4	−20.2 ± 8.1	−20.9 ± 6.7	−21.3 ± 6.5	−21.5 ± 9.0	−21.3 ± 7.4
9	−23.3 ± 7.9	−22.8 ± 8.5	−24.9 ± 5.9	−23.7 ± 7.5	−24.9 ± 7.2	−23.9 ± 6.5	−24.4 ± 8.5	−24.4 ± 7.4
10	−24.7 ± 8.6	−21.6 ± 7.1	−19.7 ± 9.7	−22.0 ± 8.7	−25.4 ± 10.4	−27.8 ± 8.7	−21.8 ± 9.8	−25.0 ± 9.8
11	−26.4 ± 9.2	−30.9 ± 9.0	−24.7 ± 8.5	−27.3 ± 9.2	−33.2 ± 10.1	−32.7 ± 11.4	−30.1 ± 11.6	−32.0 ± 11.0
12	−25.0 ± 11.6	−24.9 ± 10.9	−26.0 ± 8.5	−25.3 ± 10.3	−30.5 ± 9.2	−32.8 ± 11.2	−32.1 ± 9.2	−31.8 ± 9.8
13	−29.8 ± 7.1	−30.2 ± 8.8	−26.9 ± 10.5	−29.0 ± 8.9	−30.3 ± 8.9	−33.9 ± 10.9	−33.0 ± 11.9	−32.4 ± 10.6
14	−17.7 ± 5.7	−15.7 ± 6.1	−16.1 ± 4.4	−16.5 ± 5.4	−17.2 ± 7.6	−15.2 ± 5.2	−17.6 ± 4.8	−16.7 ± 6.0
15	−27.7 ± 9.9	−24.3 ± 11.4	−17.9 ± 9.0	−23.3 ± 10.8	−24.7 ± 11.1	−17.9 ± 9.7	−21.7 ± 10.0	−21.4 ± 10.5
16	−20.1 ± 7.6	−15.9 ± 6.9	−17.2 ± 5.5	−17.8 ± 6.9	−17.8 ± 10.1	−13.0 ± 7.8	−15.8 ± 7.6	−15.5 ± 8.7
17	−28.0 ± 5.5	−23.1 ± 5.2	−22.5 ± 5.1	−24.5 ± 5.8	−23.9 ± 6.5	−22.1 ± 5.4	−24.0 ± 4.2	−23.3 ± 5.4
Basal	−22.2 ± 2.9	−21.0 ± 3.8	−22.7 ± 4.0	−21.9 ± 3.6	−24.0 ± 3.7	−25.5 ± 3.7	−22.7 ± 4.7	−24.0 ± 4.2
Mid	−22.8 ± 4.4	−23.5 ± 4.6	−23.4 ± 3.6	−23.2 ± 4.2	−27.2 ± 3.6	−27.0 ± 4.2	−25.6 ± 4.5	−26.6 ± 4.1
Apical	−23.8 ± 4.2	−21.5 ± 4.5	−19.5 ± 4.2	−21.6 ± 4.6	−22.5 ± 5.5	−20.0 ± 5.0	−22.0 ± 4.0	−21.5 ± 4.9
Anterior	−20.8 ± 3.3	−20.5 ± 4.1	−21.3 ± 3.8	−20.9 ± 3.7	−24.4 ± 4.0	−23.4 ± 4.3	−22.3 ± 3.7	−23.4 ± 4.1
Septum	−20.1 ± 3.2	−18.3 ± 3.4	−19.2 ± 3.4	−19.2 ± 3.4	−20.7 ± 3.3	−20.7 ± 3.3	−19.0 ± 4.7	−20.1 ± 3.9
Inferior	−23.4 ± 4.3	−21.4 ± 3.9	−20.9 ± 3.9	−21.9 ± 4.1	−24.4 ± 6.9	−23.8 ± 3.7	−22.1 ± 4.2	−23.4 ± 5.1
Lateral	−26.4 ± 3.8	−27.1 ± 4.3	−26.4 ± 3.1	−26.6 ± 3.8	−29.4 ± 3.0	−30.1 ± 4.0	−29.9 ± 4.7	−29.8 ± 3.9

*Data are expressed as mean ± SD. 1, basal anterior; 2, basal anterolateral; 3, basal inferoseptal; 4, basal inferior; 5, basal inferolateral; 6, basal anterolateral; 7, mid anterior; 8, mid anteroseptal; 9, mid inferoseptal; 10, mid inferior; 11, mid inferolateral; 12, mid anterolateral; 13, apical anterior; 14, apical septal; 15, apical inferior; 16, apical lateral; 17, apex.*

RV *E*_ll_ showed no significant differences among three age groups (-30.0 ± 6.1, −29.1 ± 6.7, and −28.8 ± 5.0%, *P* = 0.559). *E*_ll_ values in males and females were −28.5 ± 6.4 and −30.1 ± 5.5%, but not statistically significant (*P* = 0.104).

### Reproducibility

The intra- and inter-observer variability results were given in **Table 9**. In Bland–Altman analyses, LV *E*_cc_ had the best intra-observer agreement (bias, −0.04 ± 0.72; 95% CI, −1.48 to 1.41), and LV *E*_ll_ had the best inter-observer agreement (bias, −0.07 ± 0.77; 95% CI, −1.61 to 1.47). All parameters had an intra- and inter- observer ICC > 0.89, except for LV *E*_rr_ with intra- (0.793) and inter- (0.832) observer.

**Table 9:** Intra-observer and inter-observer variability.

Variable	Variability	Mean bias ± SD	Limits of agreement	Coefficient of variation (%)	ICC (95% CI)
LA *E*_ll_	Intra-observer	−0.41 ± 1.24	−2.88 to 2.07	2.43	0.994 (0.985, 0.998)
	Inter-observer	3.97 ± 4.05	−4.12 to 12.06	14.85	0.929 (0.820, 0.972)
LV *E*_ll_	Intra-observer	−0.22 ± 0.89	−2.00 to 1.56	3.32	0.966 (0.913, 0.986)
	Inter-observer	−0.07 ± 0.77	−1.61 to 1.47	2.88	0.969 (0.921, 0.988)
LV *E*_cc_	Intra-observer	−0.04 ± 0.72	−1.48 to 1.41	2.15	0.990 (0.975, 0.996)
	Inter-observer	−0.44 ± 1.31	−2.18 to 3.05	4.03	0.960 (0.898, 0.984)
LV *E*_rr_	Intra-observer	−1.02 ± 14.44	−29.89 to 27.85	11.28	0.793 (0.477, 0.918)
	Inter-observer	4.22 ± 15.04	−25.85 to 34.29	12.54	0.832 (0.576, 0.934)
RV *E*_ll_	Intra-observer	−0.54 ± 1.66	−3.86 to 2.78	3.92	0.975 (0.937, 0.990)
	Inter-observer	−4.43 ± 3.18	−10.8 to 1.94	14.85	0.891 (0.726, 0.957)

*LA, left atrium; LV, left ventricle; RV, right ventricle; E_ll_, longitudinal strain; E_cc_, circumferential strain; E_rr_, radial strain; ICC, intra-class coefficient; CI, confidence interval.*

## Discussion

To the best of our knowledge, this is the largest prospective study to date that quantifies global, segmental and regional strain and strain rates of healthy Chinese across a broad age range. Based on results obtained using the CMR feature tracking technique, our study demonstrates (i) higher magnitudes of longitudinal and circumferential LA and LV strain in females; (ii) regional variations in longitudinal and circumferential strains, with higher strain values in the lateral LV territories and RV free wall compared to the septal area; and (iii) independent associations between age and LA and LV global circumferential and longitudinal strains; in addition (iv) it provides quantitative ranges of LA, LV, and RV strain in healthy Chinese stratified into different age groups.

### Normal Ranges of Strain

CMR-FT, a technique analogous to echocardiographic speckle tracking, has proven to be a feasible and reproducible approach for quantifying LA dynamics in terms of strain and strain rate (Kowallick et al., 2014, 2015b). Corresponding imaging biomarkers are increasingly recognized as having the potential to predict outcomes in a variety of cardiovascular disease states (Kowallick et al., 2014, 2015a; Inoue et al., 2015; Dick et al., 2017). The basic function reflected by LA strain depends on the use of either the QRS complex (R–R gating) or the P wave at the initiation of the calculation. When the R wave is used, as in this study, the first peak between the R and T waves corresponds to reservoir function (Pathan et al., 2017). Reservoir strain of LA derived from CMR-FT in this study included 32.8 ± 9.2% for longitudinal and 40.3 ± 13.4% for circumferential strain. The longitudinal strain value was lower than 46 ± 13% reported by Dick et al. (2017) from 25 healthy subjects. The lower LA volume in our subjects may explain the difference in *E*_ll_ values, since it has been reported that deformation parameters from CMR-FT for atrial reservoir functions are strongly related to volumetric indexes (Kowallick et al., 2014).

The LV myocardial *E*_ll_ and *E*_cc_ values obtained in our study were similar to the normal ranges of a recently published systematic review and meta-analysis (Vo et al., 2018). Andre et al. (2015) reported FT-derived LV longitudinal strain and circumferential strain values of −21.6 ± 3.2 and −21.3 ± 3.3% in 150 healthy volunteers at 1.5T (Achieva, Philips Medical Systems), which is concordant with our results. Taylor (Taylor et al., 2015), using CMR-FT at 1.5T (Magnetom Avanto, Siemens), reported values of −21.3 ± 4.8 and −26.1 ± 3.8% derived from a group of 100 individuals comprising 10 men and women in each of 5 age deciles. The values are also in close agreement with our study. Relative greater radial strain exhibited in this study than previously results reported in (Andre et al., 2015; Taylor et al., 2015; Vo et al., 2018). It may be partly explained by that in our study, papillary muscles were included in the endocardial borders, which would have resulted in greater LV volume estimates with the consequent higher values for myocardial radial strain. Moreover, feature tracking imaging (FTI) algorithms inherently depend on image quality and endocardial border definition. And the large standard deviation of radial strain (up to 19.4% in this study) may suggest limitation for the present FTI algorithms in evaluating radial strain and advanced algorithms may be warranted. Our results showed regional variations that LV longitudinal and circumferential strain, and strain rate were highest in the lateral walls and lowest in the septum. Peak circumferential strain was lower in the mid-cavity than at the base or apex, which is consistent with results in Taylor et al. (2015).

The RV longitudinal strain measured in our study was consistent with that of Levy et al. (2014) who reported a range of −20.8 to −34.1% (mean, −29.0%; 95% CI, −31.5 to −26.5%) based on a meta-analysis of two-dimensional speckle tracking echocardiographic-derived right ventricular strain in children. Our values for RV longitudinal strain were lower than those of Truong et al. (2017) and Liu et al. (2018), both post-processing with Circle Cardiovascular Imaging Tissue Tracking software. RV strains were obtained from 50 consecutive patients with no identified cardiac pathology (-22.1 ± 3.5%; ages 4–81 years; median age, 32 years) in Truong (Truong et al., 2017), and 100 healthy subjects containing 10 males and 10 females from each decade (-21.9 ± 3.24%; ages 20–70 years) (Liu et al., 2018). CMR-FT is based on the features at the myocardial boundary voxels and RV strain assessment software only measures endocardial strain, while Truong and Liu used both endocardial and epicardial borders to determine the myocardial deformable model. Secondly, RV strain assessment in our study included the septal values, as CMR-FT utilizes a LV tracking program, and Truong et al. (2017) only assessed the free wall without the inter-ventricular septum. More importantly, compared to CMR tissue tracking (CMR TT) based on the myocardium (Truong et al., 2017), manual intervention was needed to correct inaccurate tracking results in the feature tracking software, which could potentially introduce inconsistencies arising from image noise and the complex anatomical structure along the boundary. It would seem reasonable that differences in feature tracking software and strain assessment methods, a very thin free wall and presence of heavy trabeculations, variability among subjects, all combined to produce different values of RV longitudinal strain.

### Gender and Age Specific of Cardiac Deformation

Conflicts remain regarding the effects of gender and age on cardiac deformation. Vo et al. (2018) showed no association of LV *E*_ll_, *E*_rr_ and RV *E*_ll_ with age, gender, software, field strength, sequence, LVEF or LV size. However, most published studies, such those as by Lawton et al. (2011), Augustine et al. (2013), and Taylor et al. (2015) reported greater strain in females, which is consistent with the trend in our results.

The LA *E*_ll_, *E*_cc_ and LV *E*_cc_, *E*_rr_ exhibited age dependency, although the correlation was weak. Systolic strain declined with increasing age. Higher correlation of LA strain with age compared to LV strain suggests greater clinical impact of age on LA strain. In contrast, age-related LV stiffness associated with a decline in diastolic function could be compensated for by increases in systolic wall thickening, thereby explaining the increase in radial strain with age. While our findings conflict with the result in Taylor et al. (2015), who reported an age-related increase in circumferential strain, our findings concur with those from Kuznetsova et al. (2008) and Dalen et al. (2010) who reported a decline in longitudinal strain associated with age. The discordance may be due to complexities in the course of aging rather than simply age and gender, hence the statistically significant but weak effects of age in our results.

Finally, we did not observe a significant association between age and RV longitudinal strain. This is consistent with the results from previous studies using speckle tracking echocardiography (Levy et al., 2014) and CMR tissue tracking (Truong et al., 2017; Liu et al., 2018; Vo et al., 2018).

### Reproducibility

In this study, acceptable intra- and inter-observer agreement was found for peak systolic strain of LA, LV, and RV. Reproducibility obtained for LV *E*_cc_ was the best, followed by *E*_ll_ and then *E*_rr_. This is consistent with the findings of Taylor et al. (2015). It suggested that FTI allowed for reproducible quantification of systolic strains. And validation of LV *E*_rr_ is more challenging as this is generally less accurately quantified by all deformation algorithms.

### Clinical Perspective

CMR-FT derived wall motion assessment reliably quantifies LA, LV, and RV strain and SR from standard SSFP cine images. It seems a promising approach for the study of physiology in health and disease states. CMR-FT based global and segmental deformation quantification helps early detecting of subclinical myocardial dysfunction, monitoring the progress and predicting the outcome of the disease, such as heart failure with preserved ejection fraction (Zhong et al., 2013). Quantitative assessment of systolic right ventricular myocardial deformation can be used as a more quantitative tool to measure RV function in diseases such as repaired tetralogy of Fallot (Zhong et al., 2012) and pulmonary arterial hypertension (Leng et al., 2016).

### Limitations of Study

Given the higher prevalence of risk factors such as diabetes mellitus, atherosclerosis, hypertension, and hyperlipidemia among the very elderly subjects, future studies may be required to focus on the normal ranges for these groups after stratification by risk factors. Other risk factors such as smoking, blood pressure, alcohol consumption are also missing in the present study, and how they will affect the strain values needs further investigation. Right atrial deformation was excluded from this analysis, as border tracking of the right atrium is known to be technically challenging owing to thin walls and the potential for morphological variations. This may be a contributing factor to the current lack of consensus on the clinical value of right atrial strain analysis.

## Author Contributions

XM, ML, and LZ conceived and designed the study. JP, XZ, LZ, ZF, ZW, HC, SL, R-ST, and AK performed the experiments. JP wrote the paper. XM, ML, LZ, ZF, XZ, and JA reviewed and edited the manuscript. All authors read and approved the manuscript.

## Conflict of Interest Statement

The authors declare that the research was conducted in the absence of any commercial or financial relationships that could be construed as a potential conflict of interest.
